# Genome-Wide Association Analysis and Genomic Prediction of Thyroglobulin Plasma Levels

**DOI:** 10.3390/ijms23042173

**Published:** 2022-02-16

**Authors:** Nikolina Pleić, Mirjana Babić Leko, Ivana Gunjača, Thibaud Boutin, Vesela Torlak, Antonela Matana, Ante Punda, Ozren Polašek, Caroline Hayward, Tatijana Zemunik

**Affiliations:** 1 Department of Medical Biology, School of Medicine, University of Split, Šoltanska 2, 21000 Split, Croatia; npleic@mefst.hr (N.P.); mbabic@mefst.hr (M.B.L.); igunjaca@mefst.hr (I.G.); antonela.matana@gmail.com (A.M.); 2 MRC Human Genetics Unit, Institute of Genetics and Cancer, University of Edinburgh, Edinburgh EH4 2XU, UK; tboutin@ed.ac.uk (T.B.); caroline.hayward@ed.ac.uk (C.H.); 3 Department of Nuclear Medicine, University Hospital Split, Spinčićeva 1, 21000 Split, Croatia; veselakbsplit@yahoo.com (V.T.); ante.punda@mefst.hr (A.P.); 4 Department of Public Health, School of Medicine, University of Split, Šoltanska 2, 21000 Split, Croatia; opolasek@gmail.com

**Keywords:** genome-wide association study, thyroglobulin, thyroid, ST6GAL1, LMM, BSLMM

## Abstract

Thyroglobulin (Tg) is an iodoglycoprotein produced by thyroid follicular cells which acts as an essential substrate for thyroid hormone synthesis. To date, only one genome-wide association study (GWAS) of plasma Tg levels has been performed by our research group. Utilizing recent advancements in computation and modeling, we apply a Bayesian approach to the probabilistic inference of the genetic architecture of Tg. We fitted a Bayesian sparse linear mixed model (BSLMM) and a frequentist linear mixed model (LMM) of 7,289,083 variants in 1096 healthy European-ancestry participants of the Croatian Biobank. Meta-analysis with two independent cohorts (total n = 2109) identified 83 genome-wide significant single nucleotide polymorphisms (SNPs) within the *ST6GAL1* gene ($p<5\times 10^{-8}$). BSLMM revealed additional association signals on chromosomes 1, 8, 10, and 14. For *ST6GAL1* and the newly uncovered genes, we provide physiological and pathophysiological explanations of how their expression could be associated with variations in plasma Tg levels. We found that the SNP-heritability of Tg is 17% and that 52% of this variation is due to a small number of 16 variants that have a major effect on Tg levels. Our results suggest that the genetic architecture of plasma Tg is not polygenic, but influenced by a few genes with major effects.

## 1. Introduction

Thyroglobulin (Tg) is the most abundant protein produced by the thyroid gland. This 660 kDa iodoglycoprotein serves as a storehouse of thyroid hormones since Tg proteolysis releases thyroxine (T4) and triiodothyronine (T3) [1]. Tg is synthesized in thyrocytes. Following the post-translational modifications occurring in the rough endoplasmic reticulum and the Golgi apparatus, Tg is released into the follicular lumen where Tg iodination and hormone production occur [2,3]. Mature Tg is then transferred back to the thyrocytes by endocytosis. In the thyrocytes, Tg proteolysis occurs and thyroid hormones are released into the bloodstream on the basolateral membrane [1]. Some portion of intact Tg (mostly poorly sialylated or iodinated) can be transferred by transcytosis from the follicular lumen to the bloodstream [4]. In addition to transcytosis, Tg can be released into the blood from disrupted follicles. Moreover, plasma Tg levels are increased in thyroid pathology, and plasma Tg levels have been shown to correlate with thyroid mass [5]. A twin study showed that the observed variability in serum Tg levels has a strong genetic component [6].

Approximately 10% of Tg molecular mass is glycosylated [7]. The glycosylation of Tg is crucial for the synthesis of thyroid hormones because it has been shown that unglycosylated Tg has no potential for the synthesis of thyroid hormones [8]. Glycosylation is also important for Tg folding, iodination, trafficking and immunoreactivity [1]. Sialylation is a late post-translational modification of Tg that occurs in the Golgi apparatus [1]. ST6GAL1 (β-galactoside α-2,6-sialyltransferase), also known as sialyltransferase 1, catalyzes the addition of α-2,6 bound sialic acid to N-glycosylated proteins [9]. It is involved in the sialylation of Tg since α-2,6 bound sialic acid residues are detected at Tg [10,11]. Both ST6GAL1 mRNA [12,13,14] and protein [13,14] were detected in the thyroid gland. This membrane-bound enzyme is mainly found in the Golgi apparatus [15]. Sialylation is important for many Tg functions: immunoreactivity, autoregulation, and recycling. The desialylation of Tg increases its immunoreactivity [16,17]. In addition, poorly iodinated or sialylated Tg has a higher potential to trigger Tg-mediated signaling [18]. Sialylation also affects Tg recycling because it is important for binding Tg to its transmembrane receptor [17]. The importance of Tg sialylation for its proper functioning is evident from the case of a patient with congenital goiter with hypothyroidism. This patient had severely hyposialylated Tg and insufficient α-2,6 sialyltransferase activity [10].

Our recent genome-wide association study (GWAS) showed an association of 16 variants within the *ST6GAL1* gene with plasma Tg levels in healthy individuals [19]. This was the first GWAS to investigate genes associated with plasma Tg levels. The linear mixed model (LMM) used in our study has become a standard for genome-wide association mapping because it efficiently controls for both population structure and relatedness among individuals. However, LMMs as well as other frequentist methods only test one single nucleotide polymorphism (SNP) at a time. On the other hand, methods that relate phenotypic variation to multiple genetic variants simultaneously could further increase the power to detect causal variants. Multiple SNP modeling extensions of the standard LMM have been proposed from a Bayesian perspective by considering alternative prior distributions on the genetic effects. In the current study, we included an additional 1096 individuals and conducted association mapping using both frequentist and Bayesian approaches, as well as SNP heritability estimation and genomic prediction using Bayesian approaches. We aimed to replicate the significant findings to further confirm the association of the *ST6GAL1* gene with plasma Tg levels in healthy individuals. Additionally, we sought to elucidate the genetic architecture of Tg by using Bayesian multi-SNP approaches. Finally, we meta-analyzed our new GWA results with our previously published GWA results in a combined dataset of 2190 individuals. The outcome of such a comprehensive approach will be the generation of new knowledge on the genetic background of Tg that will lead to a better understanding of the biological pathways related to thyroid function.

## 2. Results

### 2.1. Genome-Wide Association Analyses

In the new LMM association analysis, a total of 18 SNPs reached genome-wide significance. Of the significantly associated SNPs, 15 were located within the *ST6GAL1* gene on chromosome 3, and 3 were located within the *PDPN* gene on chromosome 1 (Table 1). Among the 15 SNPs within the *ST6GAL1* gene that reached genome-wide significance, 11 were replications of our previously published discovery phase genome-wide significant results. The other five genome-wide significant variants from the discovery phase were also replicated at the $1\times 10^{-4}$ *p*-value threshold (Supplementary Table S4).

**Figure 1:**
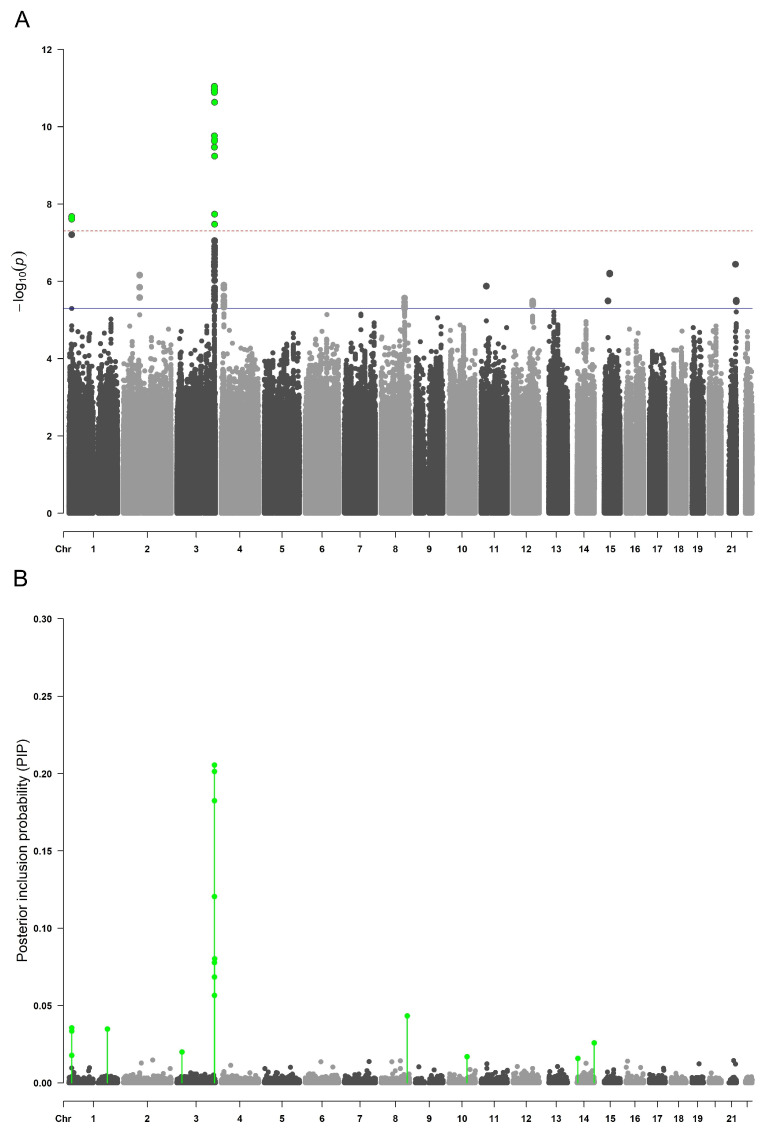
Manhattan plots of single-SNP and multi-SNP association mapping in cohorts Korcula2 and Korcula3. (**A**) Manhattan plot of single-SNP LMM analysis. The x axis represents the chromosomal position of SNPs and the y axis represents their $-\log_{10}$(*p*-values) obtained by the LMM analysis. Each dot on the Manhattan plot signifies an SNP. Because the strongest associations have the smallest *p*-values (e.g., $10^{-12}$), their negative logarithms will be the greatest (e.g., 12). The red horizontal line indicates the genome-wide significance threshold ($p=5\times 10^{-8}$), while the blue horizontal line indicates the suggestive threshold of significance ($p=5\times 10^{-6}$). (**B**) Manhattan plot of multi-SNP BSLMM analysis. The x axis represents the chromosomal position of SNPs, and the y axis represents their posterior inclusion probabilities (PIPs) obtained by the BSLMM analysis.

In the BSLMM association analysis, 16 SNPs were identified as having a major sparse effect on plasma Tg levels and these variants were estimated to have a sparse effect in ≥1.6% of BSLMM chain iterations (i.e., posterior inclusion probability (PIP) ≥ 0.016) (Supplementary Table S3). Moreover, the top four SNPs were identified as having a sparse effect on Tg levels in more than 10% of chain iterations (PIP > 0.1) and all were located within the *ST6GAL1* gene. There was a complete overlap in the significant results identified in single-SNP LMM association analysis and multi-SNP BSLMM analysis for the variants located within the *ST6GAL1* gene on chromosome 3 and *PDPN* gene on chromosome 1 (Table 1). The BSLMM approach uncovered additional association signals on chromosome 8 (rs10283166—*PVT1* gene intron variant), chromosome 14 (rs35862113—*MARK3* gene intron variant, rs61972442—*OR6J1* gene intron variant), chromosome 3 (rs1631354—*RARB* gene intron variant), and chromosome 10 (rs11202702—*RNLS* gene intron variant). Results from the single-SNP association analysis (LMM) and the multi-SNP association analysis (BSLMM) are plotted in parallel in Manhattan plots in Figure 1.

### 2.2. SNP Heritability Estimation

In our previous work [19], the top rs4012172 SNP was estimated to explain 3.19% of the variance in Tg levels. In the current study, we estimated the proportion of variance in phenotypes explained by all available genotypes (PVE) or the “chip heritability”, as well as the proportion of genetic variance explained by variants with major effect (PGE). The PVE estimate from the BSLMM with 7,289,083 SNPs indicated that 17% of the variation in plasma Tg levels was explained by all available genotypes and that 52% (PGE) of this variation was due to 16 SNPs with relatively large phenotypic effects. These results describe the genetic architecture of plasma Tg and imply that it is not purely polygenic but rather favors the sparse assumption on the variant effects. Means, medians and 95% equal tail posterior probability intervals (95% ETPPIs) of the hyperparameters estimated from the BSLMM are reported in Supplementary Table S5.

### 2.3. Genetic Prediction of Thyroglobulin Levels (Polygenic Score PGS Analysis)

To measure the prediction performance, we calculated the correlation coefficient of predicted and observed values in the test data. Keeping in mind that the PVE was estimated to be 0.17 by the BSLMM, 0.17 was considered as a theoretical upper bound for the accuracy of the predictive model. The Pearson’s correlation coefficient was equal to −0.05 (95% CI [−0.1, 0.0004]) with a *p*-value of 0.052. This result implies that we have constructed a genomic predictor of plasma Tg levels which, with the inclusion of additional training and test data, is expected to pass the 5% statistical significance threshold.

### 2.4. Meta-Analysis

**Figure 2:**
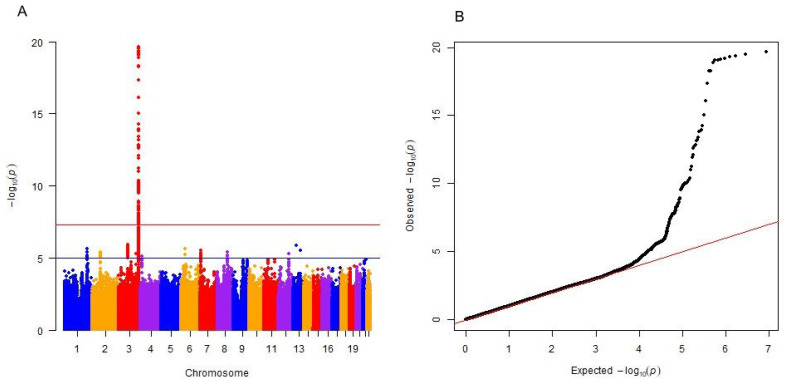
Manhattan plot and quantile–quantile (Q-Q) plot of the meta-analysis results for thyroglobulin (Tg) levels. (**A**) Manhattan plot of single nucleotide polymorphisms (SNP) for Tg levels. The x axis represents the chromosomal position of SNPs and the y axis represents their $-\log_{10}$(*p*-values) obtained by combined analysis. Each dot on the Manhattan plot signifies an SNP. The red horizontal line indicates the genome-wide significance threshold (*p* = $5\times 10^{-8}$), while the blue horizontal line indicates the suggestive threshold of significance (*p* = $5\times 10^{-6}$). (**B**) In the Q-Q plot, we see a strong deviation from the null distribution (the distribution of *p*-values under the null hypothesis of no true association is indicated by the red line).

**Figure 3:**
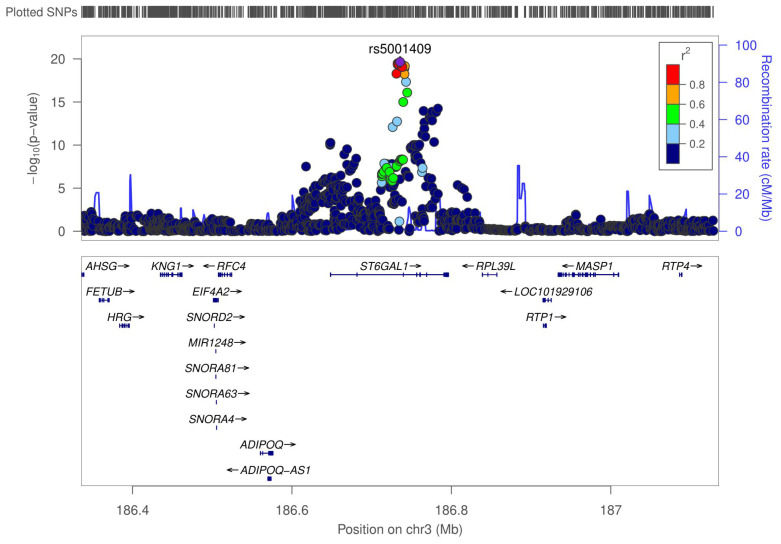
Regional association plot of the *ST6GAL1* region. The most significant SNP (rs5001409) is shown in purple. The colors of the circles denote their correlations (LD $r^{2}$) with the top SNP (lead SNP in purple, high LD SNPs with $r^{2}\geq 0.8$ in red, orange for $0.8>r^{2}\geq 0.6$, green for $0.6>r^{2}\geq 0.4$, light blue for $0.4>r^{2}\geq 0.2$ and dark blue for $r^{2}<0.2$). The figure was generated using the LocusZoom tool [20].

To attain the largest available sample size for this study, the discovery and replication datasets were meta-analyzed in order to uncover additional signals hidden in the separated discovery and replication analyses due to a lack of power. There was little evidence for population stratification at the replication-level ($\lambda_{Korcula2\&3}=1.004$) or meta-analysis level (λ=1.029). In the meta-analysis phase, 83 SNPs within the *ST6GAL1* gene on chromosome 3 reached genome-wide significance (Figure 2 and Supplementary Table S6). The most significant SNP was rs5001409 (*p* = $1.85\times 10^{-20}$). The regional association plot of the *ST6GAL1* region is shown in Figure 3. The minor C allele (MAF = 0.38) of the rs5001409 was associated with lower Tg levels (β = −0.297, SE = 0.03). Effect sizes were in the same direction in all datasets. The forest plot of the effect sizes is shown in Supplementary Figure S1.

### 2.5. Colocalization Analysis

**Figure 4:**
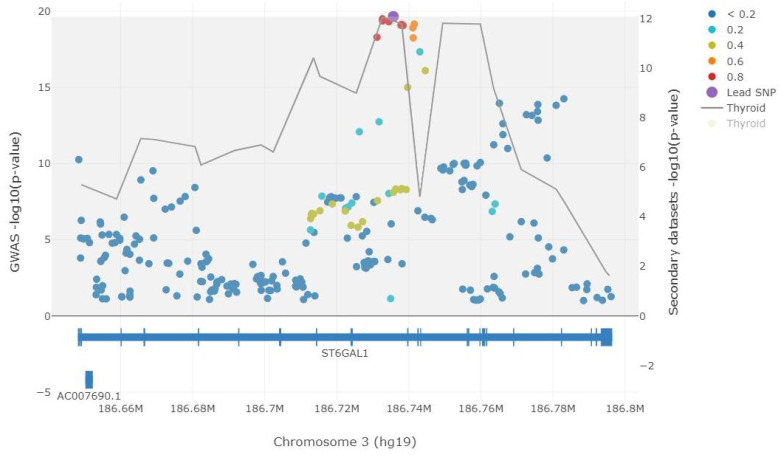
Colocalization analysis of thyroglobulin GWAS signals with eQTL signals of *ST6GAL1* gene in thyroid tissue. Filled circles represent thyroglobulin GWAS $-\log_{10}$(*p*-values) (left *y* axis). The rs5001409 SNP was defined as the lead SNP and is presented in purple. The LD information is similar to LocusZoom. The LD information was computed from the European 1000 Genomes subset (phase 1, version 3) [21] in reference to the lead SNP. The gray line represents the eQTL signals and traces the lowest *p*-value (right y axis, showing $-\log_{10}$(*p*-values)). Gene track information is from GENCODE v19 (hg19 coordinates). The figure was generated using the LocusFocus tool [22].

Our analysis supports a strong colocalization of GWAS signals with eQTLs of the *ST6GAL1* gene in thyroid tissue with an SS *p*-value of $1\times 10^{-7}$. The colocalization analysis is visualized in Figure 4. According to the GTEx portal, the most significantly associated SNP, rs5001409, was also strongly associated with the expression of the *ST6GAL1* gene in the thyroid tissue ($p=1.7\times 10^{-18}$). The association is visualized in the violin plot (Supplementary Figure S2). A normalized effect size (NES) is defined as the slope of the linear regression and is computed as the effect of the alternative allele (C allele) relative to the reference allele (A allele) in the human reference genome (i.e., the eQTL effect allele is the ALT allele). The NES of the C allele at rs5001409 was −0.33, while the median normalized expression of the *ST6GAL1* gene was 0.1952 for genotype AA, −0.0174 for genotype AC and −0.5219 for genotype CC.

## 3. Discussion

This study confirmed the results of our recent discovery GWAS on the association of the *ST6GAL1* gene with Tg plasma levels in healthy individuals [19]. In the meta-analysis, we confirmed 16 variants within the *ST6GAL1* gene previously associated with plasma Tg levels [19] and detected an additional 67 variants within the *ST6GAL1* gene that were associated with plasma Tg levels. The strongest association with plasma Tg levels was observed for the *ST6GAL1* gene rs5001409 SNP ($p=1.85\times 10^{-20}$). The C allele of this polymorphism was associated with lower plasma Tg levels. The highest expression of the *ST6GAL1* gene was found in the liver, lymph node, spleen, thyroid, and prostate tissue [23]. According to the GTEx portal, the strongest eQTL signals for the lower expression of the *ST6GAL1* gene in thyroid tissue are rs967367, rs3821819, rs10937280, rs17776120 and our top SNP, rs5001409, with an expression *p*-value of $1.7\times 10^{-18}$. The top six eQTL signals were also in the top seven signals associated with lower Tg levels in our meta-analysis. Additionally, these SNPs are in high LD with our top rs5001409 variant. This overlap was further confirmed by our colocalization analysis. We offer several explanations of how a decreased *ST6GAL1* expression may be associated with decreased plasma Tg levels. The first possibility is the association of ST6GAL1 and Tg via the Wnt/β-catenin signaling pathway. ST6GAL1 activates the Wnt/β-catenin signaling pathway through the PI3K/Akt/GSK-3β signaling pathway [24]. Lower *ST6GAL1* expression leads to a lower activation of the PI3K/Akt/GSK-3β signaling pathway, resulting in the lower activation of the Wnt/β-catenin signaling pathway. Because the Wnt/β-catenin signaling pathway activates the expression of thyroid transcription factor 1 (TTF-1) [25] (a transcription factor involved in TG transcription [26]), the lower activation of this pathway leads to lower levels of TTF-1 and consequently lower Tg levels. The second possibility of ST6GAL1 and Tg association is via the thyroid-stimulating hormone (TSH) receptor. Namely, ST6GAL1 adds sialic acid to the TSH receptor [27]. The sialylation of the TSH receptor increases the level of intracellular cAMP [28] (increased concentration of intracellular cAMP means that the TSH receptor is activated and the activation of this receptor is associated with an increased expression of the TG gene). Thus, a lower *ST6GAL1* gene expression leads to lower TSH receptor sialylation and lower TSH receptor activation. The result is a lower transcription of the *TG* gene. The third possibility is the association of ST6GAL1 and Tg through Tg. Tg has autoregulatory potential and can suppress its own expression [29,30]. Sue et al. suggested that Tg that is poorly iodinated or sialylated has a higher potential to trigger Tg-mediated signaling [18] and also has a higher affinity for the asialoglycoprotein (ASGP) receptor (one of the proposed receptors that could be involved in Tg-mediated signaling) [31,32]. Thus, a lower *ST6GAL1* expression could lead to a decrease in Tg sialylation. This would result in a higher concentration of poorly sialylated Tg which has a higher potential to trigger Tg-mediated signaling. Tg-mediated signaling can suppress *TG* gene expression. The disadvantage of this explanation is that the role of ASGPR in Tg-mediated signaling has not been thoroughly investigated, and several authors have pointed out that it is necessary to further investigate the signal transduction that occurs after Tg binding to ASGPR [31,32,33]. In addition, since lower *ST6GAL1* expression could result in a higher concentration of poorly sialylated Tg, this could increase the amount of Tg in the blood. Specifically, it is known that preferentially immature Tg (desialylated or poorly sialylated) is transferred to the blood by transcytosis [19,34].

In addition to the standard frequentist approach to GWA mapping, we performed a Bayesian multi-SNP mapping by fitting a BSLMM on 7,289,083 SNPs and 1096 individuals. The multi-SNP BSLMM approach uncovered additional association signals outside of the *ST6GAL1* gene. This study showed that the T allele in rs10283166 SNP located within the intronic region of the noncoding *PVT1* gene on chromosome 8 is associated with decreased plasma Tg levels. The *PVT1* gene encodes a long noncoding RNA that has an oncogenic role in various types of cancer [35]. Zhou et al. have shown that *PVT1* can contribute to tumorigenesis in thyroid cancer [36]. Additionally, Zhou et al. have shown that the silencing of *PVT1* reduces TSH receptor expression [36]. Because increased TSH receptor activation was associated with increased *TG* gene expression, an increase in PVT1 levels would be associated with an increase in Tg levels. Given the important role of both Tg and *PVT1* in thyroid cancer, the effect of the rs10283166 SNP on *PVT1* expression should be further investigated. On chromosome 1, the G allele, A allele, and G allele within rs78946539, rs143154928, and rs12566684 SNPs, respectively, were associated with lower plasma Tg levels. These SNPs are located within the intronic region of the *PDPN* gene. According to the GTEx portal, these SNPs affected the expression of the *RP11-474O21.5* gene in the adrenal gland, but were not associated with changes in *PDPN* gene expression. The expression of both the *RP11-474O21.5* (GEPIA database [37]) and *PDPN* [38] is increased in thyroid carcinoma. An increased expression of *PDPN* has been observed in papillary thyroid carcinoma (PTC) [38], and it has been suggested that *PDPN* may be a pro-metastatic factor in PTC [38,39]. It has been suggested that the pro-metastatic activity of *PDPN* in PTC could be through the activation of the ezrin–radixin–moesin (ERM) proteins [40]. Interestingly, moesin (ERM protein) has been shown to activate the Wnt/β-catenin signaling pathway [41] whose increased activation was associated with increased TG transcription (described earlier in the text) [26].

This study showed that the T allele in rs35862113 SNP located on chromosome 14 is associated with increased plasma Tg levels. This SNP is located in the intronic region of the Microtubule Affinity Regulating Kinase 3 (*MARK3*) gene. According to the GTEx portal, this SNP was associated with a reduced *MARK3* expression in thyroid tissue. Thus, lower *MARK3* expression results in increased plasma Tg levels. The possible association of *MARK3* with Tg is through Plakophilin-2 (PKP2) since PKP2 is one of the targets of *MARK3*. The phosphorylation of PKP2 by MARK3 creates a 14–3–3 binding site [42] and it has been suggested that the phosphorylation of PKP2 by *MARK3* and subsequent binding by 14–3–3 prevents the nuclear localization of PKP2 [43]. According to Niell et al., PKP2 antagonizes Wnt/β-catenin signaling [44] (thus, it may consequently lead to lower *TG* transcription (described earlier) [26]). Additionally, this study showed that the C allele in rs1631354 SNP, located in the intronic region of retinoic acid receptor beta gene (*RARB*) on chromosome 3, is associated with increased plasma Tg levels. According to the Human Protein Atlas, RARβ expression is high in the thyroid [13,14] while RARβ expression is reduced in thyroid carcinomas [45,46]. One previous study showed that treatment with RARβ binding retinoic acid (a metabolite of vitamin A) inhibited *TG* gene expression [47] while another showed that retinoic acid treatment increased *TG* gene expression [48].

Finally, allele A in rs11202702 SNP, on chromosome 10, was associated with an increase in Tg plasma levels. According to the GTEx portal, this allele is also associated with an increase in Ankyrin repeat domain-containing protein 22 (*ANKRD22*) expression in the esophageal mucosa (although a significant association between this SNP and *ANKRD22* expression was not observed in thyroid tissue). *ANKRD22* can activate the Wnt/β-catenin signaling pathway [49] (thus, it can consequently lead to an increase in *TG* transcription (described earlier in the text) [26]). This SNP (rs11202702) is located within the intronic region of the renalase gene (*RNLS*). To date, it has not been shown whether rs11202702 SNP affects *RNLS* gene expression. RNLS can activate AKT [50] which activates the Wnt/β-catenin signaling pathway [51] (therefore, it can consequently lead to an increase in *TG* transcription [26]).

In conclusion, the use of frequentist and Bayesian methods in inferring the genetic background of plasma Tg levels led to the confirmation of our previous results and the assessment of new parameters. We performed association mapping with both single-SNP and multi-SNP approaches. The results of the multi-SNP BSLMM approach are consistent with the results of our recent frequentist GWAS that showed an association of the *ST6GAL1* gene with plasma Tg levels in healthy individuals [19]. In the meta-analysis, we increased the sample size (from 1094 to 2190 healthy individuals) and with 16 confirmed variants [19], we found an additional 67 variants within the *ST6GAL1* gene associated with plasma Tg levels. We further fine-mapped the genetic architecture of Tg by estimating the PVE, PGE, and polygenic score. We found that all available variants explained approximately 17% of the variance in Tg levels and that 52% of this variation is due to a relatively small number of 16 variants that have a major effect on Tg levels. We constructed a predictive polygenic score of plasma Tg levels. Although polygenic predictions are of little use in the clinical setting, they facilitate new experimental designs and discoveries. For example, they can be used in a newly genotyped cohort to correlate the observed phenotypic traits with the genetic prediction of another trait. This approach yields a powerful design because if there exists an association between the traits, it must be due to genetic factors since there are no shared environmental factors [52]. This approach will be the scope of our future studies investigating the genetic factors underlying thyroid function. Because the most significant association signals in our meta-analysis were associated with both lower plasma Tg levels and lower *ST6GAL1* gene expression, we offered several explanations of how a lower *ST6GAL1* gene expression may lead to a decrease in plasma Tg levels. The molecular background of the influence of *ST6GAL1* on Tg levels should be examined in vitro and in vivo. Although our data strongly suggest the existence of additional effects beyond the *ST6GAL1* gene, further studies are needed to functionally characterize these complex effects. In addition, since Tg levels are altered in various thyroid diseases, the association of the identified genes in patients with different thyroid diseases needs to be examined. Moreover, our recent study observed an increase in *ST6GAL1* in various well-differentiated thyroid carcinomas (I.G., unpublished data). Finally, the conclusion of this study is that the genetic architecture of plasma Tg is not purely polygenic, but rather sparse, i.e., influenced by a few genes with major effects.

## 4. Materials and Methods

### 4.1. Study Population

This study was performed on participants originating from two Croatian cohorts: from the mainland city of Split (CROATIA_Split) and the island of Korcula (CROATIA_Korcula), derived from the “10,001 Dalmatians project” [53], which was part of the Croatian Biobank program. Participants were recruited from the island of Korcula in three rounds and subcohorts were named CROATIA_Korcula 1, CROATIA_Korcula 2, and CROATIA_Korcula 3, each subcohort consisting of 1000 participants. We excluded participants who could have any type of thyroid disease according to anamnestic data and detailed biochemical findings. Individuals who self-reported thyroid disorder, individuals taking thyroid medication or who underwent thyroid surgery, as well as individuals with Tg, TSH, free T3 (fT3), free T4 (fT4), Tg autoantibodies (TgAb), or thyroid peroxidase antibodies (TPOAb) levels outside of the normal reference range for our population were excluded. The published discovery phase [19] included 1094 participants from CROATIA_Split and CROATIA_Korcula 1 cohorts, and in the current study, we included an additional 1096 participants from the CROATIA_Korcula 2 and CROATIA_Korcula 3 cohorts. The final number of participants in the combined dataset for the meta-analysis was 2190. The characteristics of the cohorts are shown in Table 2. Written informed consent was obtained from participants and the study protocol was approved by the Ethical board of the University of Split, School of Medicine (No: 2181-198-03-04-14-0031 and 2181-198-03-04- 19-0022).

### 4.2. Genotyping and Imputation

Genotyping platforms and quality control procedures are summarized in Supplementary Table S1. Cohorts CROATIA_Korcula 2 and CROATIA_Korcula 3 were genotyped together using a mix of Illumina genotyping platforms CNV370v1, CNV370-Quadv3, and OmniExpressExome-8v1-2_A. Quality control (QC) steps were applied to all genotyping array data. The minimum call rate was 98% for SNPs and 97% for individuals, and autosomal SNPs not in Hardy–Weinberg equilibrium (*p*-value < $1\times 10^{-6}$) were excluded. SHAPEIT v2.r873 and the Positional Burrows–Wheeler Transform (PBWT) [54] provided by the Wellcome Sanger Institute were used for phasing and imputing data into the Haplotype Reference Consortium (HRC) reference panel [55]. Additional QC was performed on imputed data. Imputed variants not in Hardy–Weinberg equilibrium (*p*-value < $1\times 10^{-6}$), with minor allele frequency (MAF) < 0.01 or with an information score < 0.4, were excluded. Sex chromosomes were not analyzed. Due to the heavy computational burden of fitting a multi-SNP approach, only variants with an information score ≥0.9 were used for the Bayesian modeling, and the compared LMM analysis. The final number of SNPs tested for association with Tg levels was 7,289,083 for both frequentist and Bayesian approaches, and 6,554,718 overlapping SNPs for the meta-analysis. Cohorts CROATIA_Korcula 2 and CROATIA_Korcula 3 were merged with an earlier genotyped CROATIA_Korcula 1 cohort and this merged dataset was used for prediction analyses. The final number of SNPs used in the estimation of hyperparameters and prediction analyses was 7,289,083.

### 4.3. Biochemical Measurements

Levels of thyroid hormones and antibodies in the plasma of participants were determined by immunoassay methods with the Liaison XL Biomedica Chemiluminescence Analyzer. Reference ranges for the study population were: Tg 0.2–50 ng/mL, TSH 0.3–3.6 mIU/L, fT3 3.39–6.47 pmol/L, fT4 10.29– 21.88 pmol/L, TgAb 5–100 IU/mL, and TPOAb levels 1–16 IU/mL. All biochemical measurements were performed in the Biochemistry Laboratory in the Department of Nuclear Medicine at the University Hospital Split.

### 4.4. Genome-Wide Association Analyses

Genome-wide association analyses in cohorts CROATIA_Split and CROATIA_Korcula 1 were performed in our previously conducted discovery GWAS [19]. We conducted a new GWAS in an independent combined dataset CROATIA_Korcula2 and CROATIA_Korcula 3 consisting of 1096 participants. For the association analysis, we considered two different approaches: the frequentist LMM and Bayesian BSLMM, both implemented using the software GEMMA 0.98.5 [56]. The phenotype used in both approaches was the same; Tg levels were firstly regressed on sex and age using R statistical software [57] and regression residuals were further quantile normalized to a standard normal distribution.

#### 4.4.1. Linear Mixed Model (LMM)

We fit a standard LMM using GEMMA 0.98.5. in the following form:

(1) y=Wα+xβ+u+ϵ

(2) $$u∼MVN_{n}\left(0,\lambda \tau^{-1}K\right)$$

(3) $$\epsilon ∼MVN_{n}\left(0,\tau^{-1}I_{n}\right)$$

where y is a vector of Tg residuals corrected for age and sex for *n* = 1096 individuals, W is a n×c matrix of covariates (fixed effects) in our case; a column of 1s, α is a *c*-vector of the intercept; x is an n-vector of marker genotypes, β is the effect size of the marker, u is an *n*- vector of random effects; ϵ is an *n*-vector of errors; $\tau^{-1}$ is the variance of the residual errors, λ is the ratio between the two variance components, K is a known n×n relatedness matrix and $I_{n}$ is an n×n identity matrix. $MVN_{n}$ denotes the *n*-dimensional multivariate normal distribution. Effect sizes represent the change in adjusted Tg levels for each additional effect allele in the genotypes of participants.

#### 4.4.2. Bayesian Framework

LMM implemented in GEMMA with Equation (1) tests the alternative hypothesis $H_{1}:\beta \neq 0$ against the null hypothesis $H_{0}:\beta =0$ for each SNP in turn. Extensions of LMM that jointly account for the effects of variants across multiple loci could further increase power to detect causal variants. Bayesian LMMs are capable of modeling all markers jointly by assuming different prior distributions on the marker effects and sampling from their posterior distribution. Bayesian models developed for the estimation of the SNP effect sizes start with a simple linear model that relates genotypes **X** to phenotypes **y**:

(4) $$y=1_{n}\mu +X\beta +\epsilon$$

(5) $$\epsilon ∼MVN_{n}\left(0,\tau^{-1}I_{n}\right)$$

where y is a vector of phenotypes measured on *n* individuals, X is a n×p matrix of genotypes measured on that same *n* individuals at *p* genetic markers, β is a *p*-vector of genetic marker effects, $1_{n}$ is an *n*-vector of 1 s, μ is a scalar of the phenotype mean, and ϵ is an *n*-vector of error terms that have variance $\tau^{-1}$. Our aim was to estimate the parameter β, that is, the effects of genetic markers, however, since the number of genetic markers *p* in our study (7,289,083) was considerably larger than the number of individuals *n* (1096), we needed to make some modeling assumptions for SNP effect sizes β. These different assumptions on the priors vary from the infinitesimal (i.e., the polygenic) model which assumes that all SNPs have a non-zero effect, to the direct opposite, the sparse model which assumes that a relatively small proportion of all variants affect the phenotype. The performance of the model depends on the underlying true genetic architecture of the studied trait. However, in general, this true genetic architecture is unknown. The most commonly used polygenic modeling approach assumes that all SNPs affect the phenotype (have a non-zero effect) with normally distributed effect sizes:

(6) $$\beta ∼N(0,\sigma_{\beta }^{2})$$

Equation (1) with the normality assumption (6) for effect sizes β yields a model referred to as the linear mixed model (LMM) for its resulting random effect term of the combined genetic effects.

#### 4.4.3. Bayesian Sparse Linear Mixed Model (BSLMM)

A more general assumption, which includes both polygenic and sparse modeling assumptions as special cases, is that the effect sizes come from a mixture of two normal distributions:

(7) $$\beta_{i}∼\pi N\left(0,\left(\sigma_{a}^{2}+\sigma_{b}^{2}\right)/p\tau \right)+\left(1-\pi \right)N\left(0,\sigma_{b}^{2}/p\tau \right)$$

where π is the proportion of SNPs with large effects, and therefore the model is interpreted under the assumption that all variants have at least a small effect, where $\sigma_{b}^{2}/p\tau$ is the variance of small effects, and $\sigma_{a}^{2}/p\tau$ is the additional variance of large effects. The resulting model is the Bayesian sparse linear mixed model (BSLMM) proposed by Zhou et al. [58]. By assuming a combination of polygenic and sparse effects for the prior distribution of effect sizes, BSLMM is capable of adapting to different genetic architectures of the studied traits. Multi-SNP association mapping in BSLMM accounts for relatedness among individuals and population stratification by including a genomic kinship matrix as a random effect term. It also accounts for linkage disequilibrium (LD) between SNPs by estimating SNP effect sizes β while controlling for other SNPs included in the model [58]. BSLMM uses a Markov chain Monte Carlo algorithm to sample from the posterior to obtain the SNP effect size β. As opposed to *p*-values from LMM, for each SNP, it outputs a posterior inclusion probability (PIP), which is the probability that the marker is associated with the trait given the data, calculated as a proportion of chain iterations in which that SNP has a large effect. SNPs that are most robustly associated with the phenotype are therefore expected to have large PIPs and these SNPs are the most probable candidates for being the functional variants affecting plasma Tg variation. We ran a BSLMM on the same dataset (1096 individuals and 7,289,083 variants) as in our primary frequentist LMM association analysis in order to compare the single-SNP and multi-SNP approaches and to possibly reduce the incidence of false positive and false negative findings. BSLMM chain was run with default 1,000,000 sampling steps and 100,000 burn-in iterations. We used the estimated PIPs from the BSLMM output for the additional fine-mapping of the genomic regions significantly associated with Tg levels in the standard LMM analysis. The *p*-values from the LMM were plotted in parallel with PIPs from the BSLMM analysis in the Manhattan plots using the R package “CMplot” [59].

### 4.5. SNP Heritability Estimation

We estimated the proportion of variance in phenotypes explained by all available genotypes (PVE), also referred to as the “chip heritability”, by assuming that the SNP effect sizes follow a mixture of two normal distributions (Equation (7)), as implemented in GEMMA BSLMM.

### 4.6. Genetic Prediction of Thyroglobulin Levels (Polygenic Score PGS Analysis)

Predicting phenotypes from genotypes for newly observed individuals can greatly aid the development of precision medicine. However, predictions require the development of statistical methods that can accurately model the polygenic architecture of the studied trait. This is achieved by constructing a polygenic score (PGS). The simplest PGS is essentially a weighted sum of genotypes across SNPs, where weights are the estimated genetic effect sizes (β) [60]. We decided to utilize the BSLMM model for genomic prediction since this method was designed for use on individual-level data and has been demonstrated to outperform several other genomic prediction methods [58]. Tg levels were firstly regressed on sex and age using the R software. Derived residuals were quantile normalized to a standard normal distribution in R before the PGS analysis. Because GEMMA requires that the input genotype file for the PGS analysis contains both training and test data, Cohorts CROATIA_Korcula 2 and CROATIA_Korcula 3 were merged with the earlier genotyped CROATIA_Korcula 1 cohort and this merged dataset was used for constructing the PGS. Sample data from the combined cohorts CROATIA_Korcula 2 and CROATIA_Korcula 3 were used as training data, and sample data from the CROATIA_Korcula 1 cohort were used as test data. A Bayesian sparse linear mixed model was then fitted on the training data and its prediction performance was evaluated by calculating the Pearson’s correlation coefficient between the predicted and observed values in the test data. The estimate of PVE for the SNPs used in the prediction analysis represents the potential upper bound for the performance of PGS [60]. Because of this, we expected that the prediction accuracy of the most efficient PGS would not exceed the estimated value of PVE.

### 4.7. Meta-Analysis

We combined our previously conducted and published GWAS results in the CROATIA_Split and CROATIA_Korcula1 cohorts with our newly conducted GWAS in the CROATIA_Korcula 2 and 3 cohorts using a fixed-effect inverse-variance weighted model. To visualize the meta-analysis results, a Manhattan plot and a quantile–quantile (Q-Q) plot were generated using the R package ‘‘qqman’’ [61]. A regional association plot for the genomic region within 500 Kb of the top hit was generated using LocusZoom software [20], and a forest plot for the most significant SNP association was generated using the R package MetABEL.

### 4.8. GTEx Project

The Genotype-Tissue Expression (GTEx) project [23] provides the scientific community with a resource to study human gene expression and regulation and its relationship with genetic variation. By analyzing global RNA expression within individual tissues and treating the expression levels of genes as quantitative traits, variations in gene expression that are highly correlated with genetic variation can be identified as expression quantitative trait loci or eQTLs. The GTEx Project database contains the analyses of mRNA levels in 49 different tissues, including thyroid tissue obtained from 574 donors with available genotype data. The data used for the analyses described in this manuscript were obtained from the GTEx portal.

### 4.9. Colocalization Analysis

Colocalization testing brings it closer to establishing causal relationships. If an SNP is significantly associated with both Tg levels and the gene’s expression (i.e., it is an expression quantitative trait locus, eQTL), then this may suggest a regulatory role of the SNP on gene expression in the pathway to Tg levels, which can also be regarded as vertical pleiotropy. Using the LocusFocus tool [22], we tested whether our meta-analysis signals were colocalized with the eQTL signals. The LocusFocus tool implements a frequentist colocalization framework—the Simple Sum (SS) developed by Gong et al. [62]. The SS is more powerful for colocalization than existing methods, particularly in regions of high linkage disequilibrium (LD) and allelic heterogeneity. The performance of SS relative to other frequently implemented Bayesian colocalization methods designed for summary-level data was documented by Gong and collaborators [62]. To perform the colocalization analysis, we integrated our meta-analysis summary statistics data with cis-eQTL data from thyroid tissue from the GTEx project v8.

## Figures and Tables

**Table 1:** SNPs passing genome-wide significance threshold ($5\times 10^{-8}$) in the single-SNP LMM analysis and their corresponding PIPs from the multi-SNP BSLMM analysis of cohorts Korcula2 and Korcula3.

SNP	Chr	Position	Gene	Ref. Allele	Effect Allele	EAF	Single-SNP LMM Analysis in Cohorts Korcula2 and Korcula3	Multi-SNP BSLMM Analysis in Cohorts Korcula2 and Korcula3
							β (*p*-Value)	β (PIP)
rs10937280	3	186738033	*ST6GAL1*	G	A	0.35	−0.31 ($9.09\times 10^{-12}$)	−0.29 (0.21)
rs5001409	3	186735690	*ST6GAL1*	A	C	0.35	−0.31 ($9.44\times 10^{-12}$)	−0.295 (0.07)
rs9863411	3	186737820	*ST6GAL1*	C	T	0.35	−0.31 ($1.06\times 10^{-11}$)	−0.283 (0.2)
rs7634389	3	186738421	*ST6GAL1*	T	C	0.35	−0.31 ($1.12\times 10^{-11}$)	−0.292 (0.08)
rs967367	3	186734466	*ST6GAL1*	G	A	0.35	−0.31 ($1.15\times 10^{-11}$)	−0.29 (0.12)
rs3821819	3	186732725	*ST6GAL1*	G	A	0.35	−0.31 ($1.31\times 10^{-11}$)	−0.292 (0.06)
rs4686838	3	186743053	*ST6GAL1*	A	G	0.45	−0.3($2.33\times 10^{-11}$)	−0.27 (0.08)
rs10212190	3	186731157	*ST6GAL1*	A	T	0.34	−0.29 ($1.73\times 10^{-10}$)	−0.28 (0.003)
rs4012172	3	186741511	*ST6GAL1*	C	T	0.36	−0.29 ($2.19\times 10^{-10}$)	−0.27 (0.0003)
rs3872724	3	186741221	*ST6GAL1*	C	T	0.37	−0.28 ($2.37\times 10^{-10}$)	−0.27 (0.001)
rs3872723	3	186741131	*ST6GAL1*	C	T	0.36	−0.28 ($3.4\times 10^{-10}$)	0 (0)
rs28674898	3	186744563	*ST6GAL1*	G	A	0.39	0.28 ($5.81\times 10^{-10}$)	−0.28 (0.003)
rs4686844	3	186765135	*ST6GAL1*	G	A	0.56	−0.25 ($1.83\times 10^{-10}$)	−0.15 (0.0007)
rs78946539	1	13921500	*PDPN*	A	G	0.04	−0.63 ($2.1\times 10^{-8}$)	−0.51 (0.03)
rs143154928	1	13921447	*PDPN*	G	A	0.04	−0.63 ($2.32\times 10^{-8}$)	−0.5 (0.03)
rs12566684	1	13922117	*PDPN*	A	G	0.04	−0.64 ($2.46\times 10^{-8}$)	−0.5 (0.02)
rs257104	3	186775807	*ST6GAL1*	G	A	0.4	0.24 ($3.33\times 10^{-8}$)	0.17 (0.002)

Statistical analyses were performed with GEMMA LMM and BSLMM. *p*-values < $5\times 10^{-8}$ are genome-wide significant. SNPs are sorted by ascending LMM p-value. BSLMM, Bayesian sparse linear mixed model; Chr, chromosome; EAF, effect allele frequency; LMM, linear mixed model; PIP; posterior inclusion probability; SNP, single nucleotide polymorphism.

**Table 2:** Characteristics of the study population.

Cohort	Split	Korcula 1	Korcula 2	Korcula 3
*n*	605	489	593	505
Women	321 (53%)	297 (61%)	328 (55.3%)	294 (58.2%)
Age	51 (39, 61)	56 (46, 67)	54 (40, 65)	54 (39, 65)
Tg	9.20 (4.80, 14.50)	10.20 (6.40, 15.70)	10.1 (5.6, 16.4)	10.6 (7.5, 16.1)

Values in the table represent median (interquartile range) or *n* (%). *n*, number of participants; Tg, thyroglobulin.

## Data Availability

Individual-level genetic and phenotypic data from CROATIA Split and Korcula cohorts are not available to outside researchers due to privacy restrictions. Complete summary statistics from the frequentist and Bayesian genome-wide association analyses are available.

## Supplementary Materials

The following supporting information can be downloaded at: https://www.mdpi.com/article/10.3390/ijms23042173/s1.

## Abbreviations

The following abbreviations are used in this manuscript:

AD, Alzheimer’s disease; ANKRD22, Ankyrin repeat domain-containing protein 22; ASGP receptor, asialoglycoprotein receptor; β, Beta coefficients; BSLMM, Bayesian sparse linear mixed model; Chr, chromosome; fT3, free T3; fT4, free T4; eQTL, expression quantitative trait locus; ERM proteins, ezrin–radixin–moesin proteins; ETPPI, equal tail posterior probability intervals; GTEx project, Genotype-Tissue Expression project; GWA, genome-wide association; GWAS, genome-wide association study; HRC, Haplotype Reference Consortium; HWE, Hardy–Weinberg equilibrium; LD, linkage disequilibrium; LMM, linear mixed model; MAF, minor allele frequency; MARK3, Microtubule affinity regulating kinase 3; NES, normalized effect size; PBWT, Positional Burrows–Wheeler Transform; PGE, proportion of genetic variance explained by variants with major effect; PGS, polygenic score; PIP; posterior inclusion probability; PKP2, Plakophilin-2; PTC, papillary thyroid carcinoma; PVE, proportion of variance in phenotypes explained; QC, quality control; Q-Q plot, quantile–quantile plot; RARB, retinoic acid receptor beta; RNLS, renalase; SNP, single nucleotide polymorphism; SS, Simple Sum; ST6GAL1, β-galactoside α-2,6-sialyltransferase; T3, triiodothyronine; T4, thyroxine; Tg, thyroglobulin; TgAb, Tg autoantibodies; TPOAb, thyroid peroxidase antibodies; TSH, thyroid-stimulating hormone; TTF-1, thyroid transcription factor 1.
