# Supplementary material for: Genome-Wide Association Analysis and Genomic Prediction of Thyroglobulin Plasma Levels
Source: Int J Mol Sci. 2022 Feb 16;23(4):2173. doi: 10.3390/ijms23042173 (PMC8876738; doi:10.3390/ijms23042173)
Supplement: Supplementary file 1 [file ijms-23-02173-s001.zip › Supplementary data.pdf]

Table S1. Study-Specific Information on Genotyping, Imputation and Quality-Control Metrics

| | Cohorts | Split 1 | Split 2 | Korcula 1 | Korcula 2&3 |
|---|---|---|---|---|---|
| Genome-wide genotyping | N individuals | 531 | 481 | 897 | 1727 |
| | Genotyping platform and SNP panel | Illumina HumanHap 370CNV QUAD Phase 1 | Illumina HumanOmni ExpressExome8v1-2_A | Illumina HumanHap 370CNV DUO Phase 1 | Illumina CNV370v1,CNV370-Quadv3, OmniExpressExome-8v1-2_A |
| | N SNPs | 351,514 | 969,919 | 346,034 | 346,034 |
| | Genotype-calling algorithm | Illumina BeadStudio V3 | Illumina BeadStudio V3 | Illumina BeadStudio V3 | Illumina Beadstudio-Gencall V3.0 |
| SNP QC (prior to imputation) | Call rate | ≥98% per SNP | ≥98% per SNP | ≥98% per SNP | ≥98% per SNP |
| | MAF | ≥1% | ≥1% | ≥1% | ≥1% (0.01% for Exome Chip markes) |
| | HWE | $p < 10^{-7}$ | $p < 10^{-7}$ | $p < 10^{-7}$ | $p < 10^{-6}$ |

| | | | | | |
|---|---|---|---|---|---|
| Sample QC (prior to imputation) | Call rate | >97% | >97% | >97% | >97% |
| Pre-phasing | Software | SHAPEIT2 | SHAPEIT2 | SHAPEIT2 | SHAPEIT2 |
| Phasing | Software | SHAPEIT2 and duoHMM | SHAPEIT2 and duoHMM | SHAPEIT2 and duoHMM | SHAPEIT v2.r873 and duoHMM |
| Imputation | Reference panel | Merged Split2 and 1000 Genomes Project | 1000 Genomes project Phase I version 3 | 1000 Genomes project Phase I version 3 | HRC.r1-1 NB: no InDels |
| | Software | IMPUTE2 | IMPUTE2 | IMPUTE2 | PBWT (Sanger server) |
| SNP QC (after imputation) | MAF | ≥1% | ≥1% | ≥1% | ≥1% |
| | HWE | $p < 10^{-7}$ | $p <10^{-7}$ | $p < 10^{-7}$ | $p < 10^{-6}$ |
| | Info score | >0.4 | >0.4 | >0.4 | >0.4 |
| NCBI build | | b37 | b37 | b37 | b37 |

HWE, Hardy-Weinberg equilibrium; MAF, minor allele frequency; N, number; QC, quality control; SNP, single nucleotide polymorphism.

Table S2. SNPs passing genome-wide significance threshold in the single-SNP linear mixed model (LMM) analysis

| **SNP** | **Chr** | **Position** | **Gene** | **Reference allele** | **Effect allele** | **EAF** | **Effect (β)** | **SE** | **p-value** |
|---|---|---|---|---|---|---|---|---|---|
| rs10937280 | 3 | 186738033 | *ST6GAL1* | G | A | 0.352 | -0.31 | 0.04 | $9.09 \times 10^{-12}$ |
| rs5001409 | 3 | 186735690 | *ST6GAL1* | A | C | 0.352 | -0.31 | 0.04 | $9.44 \times 10^{-12}$ |
| rs9863411 | 3 | 186737820 | *ST6GAL1* | C | T | 0.352 | -0.31 | 0.04 | $1.06 \times 10^{-11}$ |
| rs7634389 | 3 | 186738421 | *ST6GAL1* | T | C | 0.352 | -0.31 | 0.04 | $1.12 \times 10^{-11}$ |
| rs967367 | 3 | 186734466 | *ST6GAL1* | G | A | 0.351 | -0.31 | 0.04 | $1.15 \times 10^{-11}$ |
| rs17776120 | 3 | 186732679 | *ST6GAL1* | C | A | 0.351 | -0.31 | 0.04 | $1.2 \times 10^{-11}$ |
| rs3821819 | 3 | 186732725 | *ST6GAL1* | G | A | 0.351 | -0.31 | 0.04 | $1.31 \times 10^{-11}$ |
| rs4686838 | 3 | 186743053 | *ST6GAL1* | A | G | 0.455 | -0.295 | 0.04 | $2.33 \times 10^{-11}$ |
| rs10212190 | 3 | 186731157 | *ST6GAL1* | A | T | 0.341 | -0.294 | 0.04 | $1.73 \times 10^{-10}$ |
| rs4012172 | 3 | 186741511 | *ST6GAL1* | C | T | 0.36 | -0.289 | 0.04 | $2.19 \times 10^{-10}$ |
| rs3872724 | 3 | 186741221 | *ST6GAL1* | C | T | 0.373 | -0.284 | 0.04 | $2.37 \times 10^{-10}$ |
| rs3872723 | 3 | 186741131 | *ST6GAL1* | C | T | 0.362 | -0.285 | 0.04 | $3.4 \times 10^{-10}$ |
| rs28674898 | 3 | 186744563 | *ST6GAL1* | G | A | 0.393 | -0.281 | 0.04 | $5.81 \times 10^{-10}$ |
| rs4686844 | 3 | 186765135 | *ST6GAL1* | G | A | 0.568 | -0.251 | 0.04 | $1.83 \times 10^{-8}$ |
| rs78946539 | 1 | 13921500 | *PDPN* | A | G | 0.041 | -0.629 | 0.11 | $2.1 \times 10^{-8}$ |
| rs143154928 | 1 | 13921447 | *PDPN* | G | A | 0.038 | -0.632 | 0.11 | $2.32 \times 10^{-8}$ |

| | | | | | | | | | |
|---|---|---|---|---|---|---|---|---|---|
| rs12566684 | 1 | 13922117 | *PDPN* | A | G | 0.039 | -0.641 | 0.11 | $2.46 \times 10^{-8}$ |
| rs257104 | 3 | 186775807 | *ST6GAL1* | G | A | 0.399 | 0.246 | 0.04 | $3.33 \times 10^{-8}$ |

LMM was fitted on 7,289,083 SNPs and 1,096 individuals. Chr, chromosome; EAF, effect allele frequency; SE, standard error; SNP, single nucleotide polymorphism.

Table S3. SNPs identified as having a major sparse effect on plasma Tg levels in the multi-SNP Bayesian sparse linear mixed model (BSLMM) analysis

| **SNP** | **Chr** | **Position** | **Gene** | **eQTL gene** | **Tissue** | **PIP** |
|---|---|---|---|---|---|---|
| rs10937280 | 3 | 186738033 | *ST6GAL1* | *ST6GAL1* | Thyroid (-) | 0.206 |
| rs9863411 | 3 | 186737820 | *ST6GAL1* | *ST6GAL1* | Thyroid (-) | 0.201 |
| rs17776120 | 3 | 186732679 | *ST6GAL1* | *ST6GAL1* | Thyroid (-) | 0.182 |
| rs967367 | 3 | 186734466 | *ST6GAL1* | *ST6GAL1* | Thyroid (-) | 0.120 |
| rs4686838 | 3 | 186743053 | *ST6GAL1* | *ST6GAL1* | Thyroid (-) | 0.08 |
| rs7634389 | 3 | 186738421 | *ST6GAL1* | *ST6GAL1* | Thyroid (-) | 0.078 |
| rs5001409 | 3 | 186735690 | *ST6GAL1* | *ST6GAL1* | Thyroid (-) | 0.068 |
| rs3821819 | 3 | 186732725 | *ST6GAL1* | *ST6GAL1* | Thyroid (-) | 0.057 |
| rs10283166 | 8 | 129010909 | *PVT1* | / | / | 0.043 |
| rs143154928 | 1 | 13921447 | *PDPN* | *RP11-474O21.5* | Adrenal gland (-) | 0.036 |
| rs9787057 | 1 | 190885619 | / | / | / | 0.035 |
| rs78946539 | 1 | 13921500 | *PDPN* | / | / | 0.033 |
| rs35862113 | 14 | 103857229 | *MARK3* | *MARK3* | Thyroid (-) | 0.026 |
| rs1631354 | 3 | 25619920 | *RARB* | / | / | 0.02 |
| rs12566684 | 1 | 13922117 | *PDPN* | *RP11-474O21.5* | Adrenal gland (-) | 0.018 |
| rs11202702 | 10 | 90054268 | *RNLS* | *ANKRD22* | Esophagus - Mucosa (+) | 0.017 |

| | | | | | | |
|---|---|---|---|---|---|---|
| rs61972442 | 14 | 23109649 | *OR6J1* | / | / | 0.016 |

BSLMM was fitted on 7,289,083 SNPs and 1,096 individuals. Chr, chromosome; eQTL, expression quantitative trait locus; PIP, posterior inclusion probability; SNP, single nucleotide polymorphism.

Table S4. SNPs passing genome-wide significance threshold in the discovery phase and their effect sizes and p-values in replication and meta-analysis phases.

| SNP | Chr | Position | Reference allele | Effect allele | EAF | Discovery LMM meta-analysis in cohorts Split & Korcula1 | | Replication LMM in cohorts Korcula2 & Korcula3 | | LMM meta-analysis in cohorts Split & Korcula1 & Korcula2 & Korcula3 | |
|---|---|---|---|---|---|---|---|---|---|---|---|
| | | | | | | Effect (β) | p-value | Effect (β) | p-value | Effect (β) | p-value |
| rs4012172 | 3 | 186741511 | C | T | 0.36 | -0.3 | $1.29 \times 10^{-10}$ | -0.28 | $2.19 \times 10^{-10}$ | -0.291 | $5.25 \times 10^{-20}$ |
| rs3872723 | 3 | 186741131 | C | T | 0.36 | -0.29 | $1.49 \times 10^{-10}$ | -0.285 | $3.4 \times 10^{-10}$ | -0.29 | $9.88 \times 10^{-20}$ |
| rs4686837 | 3 | 186739677 | G | A | 0.29 | -0.3 | $2.34 \times 10^{-10}$ | -0.24 | $5.5 \times 10^{-7}$ | -0.271 | $6.99 \times 10^{-16}$ |
| rs3872724 | 3 | 186741221 | C | T | 0.37 | -0.28 | $9.84 \times 10^{-10}$ | -0.284 | $2.37 \times 10^{-10}$ | -0.283 | $4.23 \times 10^{-19}$ |
| rs10212190 | 3 | 186731157 | A | T | 0.38 | -0.28 | $1.25 \times 10^{-9}$ | -0.294 | $1.73 \times 10^{-10}$ | -0.288 | $3.84 \times 10^{-19}$ |
| rs5001409 | 3 | 186735690 | A | C | 0.38 | -0.28 | $1.38 \times 10^{-9}$ | -0.312 | $9.44 \times 10^{-12}$ | -0.297 | $1.85 \times 10^{-20}$ |
| rs17776120 | 3 | 186732679 | C | A | 0.38 | -0.28 | $1.55 \times 10^{-9}$ | -0.309 | $1.2 \times 10^{-11}$ | -0.295 | $2.62 \times 10^{-20}$ |
| rs3821819 | 3 | 186732725 | G | A | 0.38 | -0.28 | $1.9 \times 10^{-9}$ | -0.31 | $1.31 \times 10^{-11}$ | -0.294 | $3.62 \times 10^{-20}$ |
| rs967367 | 3 | 186734466 | G | A | 0.38 | -0.28 | $2.42 \times 10^{-9}$ | -0.31 | $1.15 \times 10^{-11}$ | -0.293 | $4.03 \times 10^{-20}$ |
| rs7634389 | 3 | 186738421 | T | C | 0.38 | -0.27 | $4.25 \times 10^{-9}$ | -0.31 | $1.12 \times 10^{-11}$ | -0.292 | $7.17 \times 10^{-20}$ |
| rs9863411 | 3 | 186737820 | C | T | 0.38 | -0.27 | $4.28 \times 10^{-9}$ | -0.31 | $1.06 \times 10^{-11}$ | -0.291 | $6.74 \times 10^{-20}$ |

| | | | | | | | | | | | |
|---|---|---|---|---|---|---|---|---|---|---|---|
| rs10937280 | 3 | 186738033 | G | A | 0.38 | -0.27 | $4.46 \times 10^{-9}$ | -0.31 | $9.09 \times 10^{-12}$ | -0.291 | $5.99 \times 10^{-20}$ |
| rs257099 | 3 | 186783032 | C | T | 0.36 | 0.28 | $5.18 \times 10^{-9}$ | 0.238 | $3.16 \times 10^{-7}$ | 0.26 | $6.58 \times 10^{-15}$ |
| rs6804130 | 3 | 186763561 | A | C | 0.45 | -0.25 | $7.12 \times 10^{-9}$ | -0.173 | $1.11 \times 10^{-4}$ | -0.214 | $6.9 \times 10^{-12}$ |
| rs7619989 | 3 | 186731749 | G | C | 0.38 | -0.25 | $2.57 \times 10^{-8}$ | -0.216 | $1.71 \times 10^{-6}$ | -0.23 | $1.63 \times 10^{-13}$ |
| rs10433485 | 3 | 186780866 | G | A | 0.35 | -0.24 | $4.31 \times 10^{-8}$ | -0.24 | $1.43 \times 10^{-7}$ | -0.241 | $1.81 \times 10^{-14}$ |

Statistical analyses were performed with R: GenABEL and SNPTEST for the discovery phase and GEMMA LMM for the replication phase. For the meta-analyses, the datasets were combined and analyses were performed using a fixed-effects inverse-variance weighted model in R. P-values $< 5 \times 10^{-8}$ are genome-wide significant. Chr, chromosome; EAF, effect allele frequency; LMM, linear mixed model; SNP, single nucleotide polymorphism.

Table S5. Means, medians, and 95% equal tail posterior probability intervals (95% ETPPIs) of hyperparameters estimated from the Bayesian sparse linear mixed model (BSLMM) GWA mapping in cohorts Korcula2 & Korcula3

| Hyperparameter | Mean | Median | 2.5% | 97.5% |
|---|---|---|---|---|
| h | 0.205 | 0.191 | 0.048 | 0.447 |
| PVE | 0.1696 | 0.164 | 0.0596 | 0.315 |
| rho | 0.578 | 0.589 | 0.1296 | 0.971 |
| PGE | 0.519 | 0.494 | 0.156 | 0.963 |
| pi | $2.24 \times 10^{-6}$ | $1.14 \times 10^{-6}$ | $1.71 \times 10^{-7}$ | $9.18 \times 10^{-6}$ |
| n.gamma | 15.97 | 8 | 1 | 67 |

BSLMM was fitted on 7,289,083 SNPs and 1,096 individuals. h, approximation to the proportion of phenotypic variance explained by variants (PVE); n.gamma, number of variants with major effect; pi, proportion of variants with non-zero effects; PGE, proportion of genetic variance explained by variants with major effect; PVE, proportion of phenotypic variance explained by variants; rho, approximation to the proportion of genetic variance explained by variants with major effect.

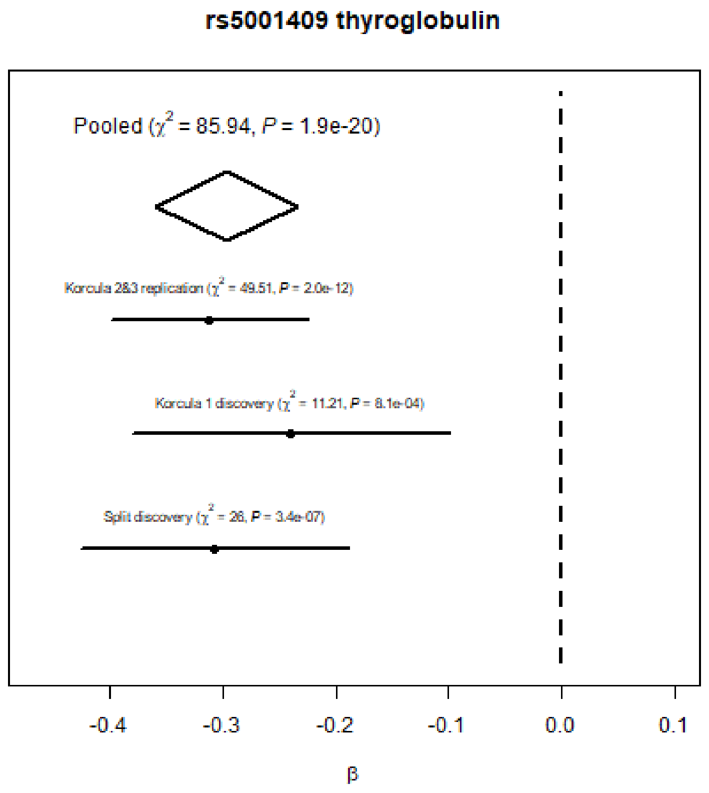


Figure S1. Forest plot of the effect sizes (β) and their 95% confidence intervals for the associations between the *ST6GAL1* gene polymorphism rs5001409 and Tg levels in the two discovery datasets (Split and Korcula 1), in the replication dataset (Korcula 2&3), and pooled (meta-analysis) effect.

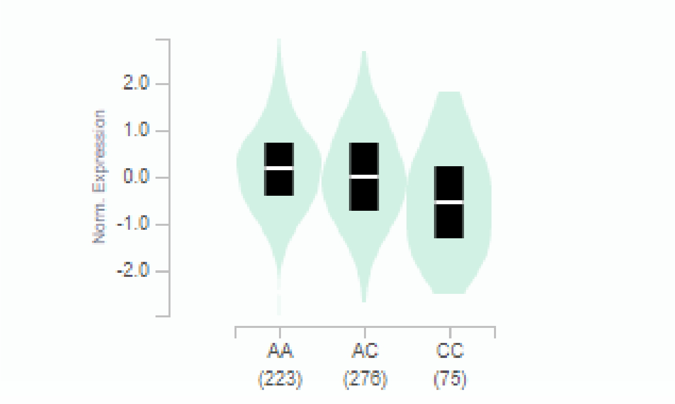


Figure S2. *ST6GAL1* expression in carriers of the rs5001409 SNP. Expression of the *ST6GAL1* gene is decreased ($p = 1.7 \times 10^{-18}$) in heterozygous and homozygous carriers of the C allele of the rs5001409 SNP associated with Tg levels. The normalized effect size (NES) of the C allele relative to the A allele is -0.33. The data and figure were obtained from the GTEx project eQTL analysis for rs5001409 SNP.
