# Supplementary material for: Genome-Wide Association Analysis and Genomic Prediction of Thyroglobulin Plasma Levels
Source: Int J Mol Sci. 2022 Feb 16;23(4):2173. doi: 10.3390/ijms23042173 (PMC8876738; doi:10.3390/ijms23042173)
Supplement: Supplementary file 1 [file ijms-23-02173-s001.zip › Supplementary Table S6.pdf]

Supplementary Table S6. Summary statistics of thyroglobulin associations for all SNPs with $p < 5 \times 10^{-8}$ in the meta-analysis

SNP	Genome context	Chromosome	Position	Reference allele	Effect allele	MAF	Info score	β (Effect)	SE	P-value
rs5001409	Intron	3	186735690	A	C	0.38	0.97	-0.297	0.03	1.85E-20
rs17776120	Intron	3	186732679	C	A	0.38	0.98	-0.295	0.03	2.62E-20
rs3821819	Intron	3	186732725	G	A	0.38	0.98	-0.294	0.03	3.62E-20
rs967367	Intron	3	186734466	G	A	0.38	0.98	-0.293	0.03	4.03E-20
rs4012172	Intron	3	186741511	C	T	0.36	0.97	-0.292	0.03	5.25E-20
rs10937280	Intron	3	186738033	G	A	0.38	0.97	-0.291	0.03	5.99E-20
rs9863411	Intron	3	186737820	C	T	0.38	0.97	-0.291	0.03	6.74E-20
rs7634389	Intron	3	186738421	T	C	0.38	0.97	-0.292	0.03	7.17E-20
rs3872723	Intron	3	186741131	C	T	0.36	0.97	-0.290	0.03	9.88E-20
rs10212190	Intron	3	186731157	A	T	0.38	0.97	-0.288	0.03	3.85E-19
rs3872724	Intron	3	186741221	C	T	0.37	0.97	-0.283	0.03	4.23E-19
rs4686838	Intron	3	186743053	A	G	0.46	0.98	-0.267	0.03	3.78E-18
rs28674898	Intron	3	186744563	G	A	0.40	0.96	-0.269	0.03	5.92E-17
rs4686837	5' UTR variant	3	186739677	G	A	0.29	1.00	-0.271	0.03	6.99E-16
rs257099	Intron	3	186783032	C	T	0.36	0.83	0.260	0.03	6.58E-15
rs4686844	Intron	3	186765135	G	A	0.40	0.93	-0.240	0.03	1.39E-14
rs257104	Intron	3	186775807	G	A	0.35	0.99	0.237	0.03	1.67E-14
rs10433485	Intron	3	186780866	G	A	0.35	0.97	-0.241	0.03	1.81E-14
rs257105	Intron	3	186775719	G	C	0.35	0.99	0.232	0.03	4.80E-14
rs9968113	Intron	3	186772620	T	A	0.34	0.97	0.233	0.03	7.67E-14
rs7619989	Intron	3	186731749	G	C	0.38	0.98	-0.231	0.03	1.63E-13
rs257103	Intron	3	186775881	C	T	0.35	0.99	0.229	0.03	1.76E-13
rs9876699	Intron	3	186766097	T	C	0.41	0.97	-0.224	0.03	2.79E-13
rs3872722	Intron	3	186726277	C	A	0.38	0.99	-0.224	0.03	7.59E-13

rs6800338	Intron	3	186766122	T	C	0.40	0.98	-0.217	0.03	1.45E-12
rs6804130	Intron	3	186763561	A	C	0.45	0.93	-0.214	0.03	6.90E-12
rs4012245	Intron	3	186767440	G	A	0.40	0.99	-0.207	0.03	1.21E-11
rs2041965	5' UTR variant	3	186648411	C	T	0.34	0.98	-0.219	0.03	3.74E-11
rs28366037	Regulatory region variant	3	186647765	C	T	0.34	0.98	-0.219	0.03	3.83E-11
rs1468906	Regulatory region variant	3	186648117	A	G	0.34	0.98	-0.218	0.03	4.32E-11
rs4012246	Intron	3	186778382	C	T	0.40	1.00	-0.201	0.03	4.96E-11
rs28366036	Regulatory region variant	3	186647763	A	C	0.34	0.98	-0.217	0.03	6.11E-11
rs2268530	Intron	3	186759850	G	A	0.39	0.98	-0.202	0.03	1.04E-10
rs4686841	Intron	3	186755743	G	A	0.39	1.00	-0.203	0.03	1.19E-10
rs4686839	Intron	3	186752571	T	C	0.40	0.98	-0.202	0.03	1.20E-10
rs4686451	Intron	3	186755759	C	T	0.39	1.00	-0.203	0.03	1.21E-10
rs6444195	Intron	3	186755344	C	T	0.39	0.99	-0.203	0.03	1.21E-10
rs9858925	Intron	3	186752308	T	A	0.39	0.99	-0.202	0.03	1.41E-10
rs4686840	Intron	3	186755620	A	G	0.39	1.00	-0.201	0.03	1.53E-10
rs6444196	Intron	3	186755882	G	A	0.40	1.00	-0.199	0.03	1.55E-10
rs9847687	Intron	3	186758930	G	C	0.40	0.99	-0.200	0.03	1.75E-10
rs2378123	Intron	3	186749659	G	C	0.39	0.98	-0.201	0.03	2.08E-10
rs3954172	Intron	3	186749415	T	G	0.39	0.98	-0.198	0.03	2.46E-10
rs3892779	Intron	3	186748887	A	T	0.39	0.98	-0.200	0.03	2.63E-10
rs4012164	Intron	3	186749444	G	A	0.39	0.99	-0.197	0.03	3.28E-10
rs9874262	Intron	3	186669005	C	T	0.48	0.93	0.199	0.03	3.33E-10
rs4012241	Intron	3	186751378	T	A	0.39	0.99	-0.196	0.03	3.70E-10
rs59688792	Intron	3	186755323	A	C	0.16	0.99	-0.259	0.04	1.21E-09
rs3887925	Intron	3	186665645	C	T	0.45	1.00	0.188	0.03	1.35E-09

rs16861533	Intron	3	186754722	G	A	0.16	0.97	-0.258	0.04	1.54E-09
rs2268536	Intron	3	186756908	T	G	0.17	0.98	-0.246	0.04	2.41E-09
rs2268533	Intron	3	186757810	G	C	0.34	0.99	-0.195	0.03	2.60E-09
rs2268534	Intron	3	186757452	T	C	0.17	0.99	-0.245	0.04	3.07E-09
rs7622288	Intron	3	186738032	C	T	0.42	0.97	0.185	0.03	3.66E-09
rs12054411	Intron	3	186736374	T	G	0.42	0.98	0.185	0.03	3.82E-09
rs58409806	Intron	3	186739266	G	T	0.42	0.97	0.185	0.03	4.21E-09
rs10212404	Intron	3	186680644	T	C	0.48	0.91	0.189	0.03	4.33E-09
rs59427408	Intron	3	186737694	C	G	0.42	0.97	0.184	0.03	4.34E-09
rs58909335	Intron	3	186754838	T	G	0.16	0.98	-0.251	0.04	4.95E-09
rs9852850	Intron	3	186735735	G	A	0.42	0.98	0.182	0.03	6.48E-09
rs6781114	Intron	3	186734447	G	A	0.40	0.97	0.184	0.03	6.75E-09
rs11707680	Intron	3	186718462	G	A	0.21	1.00	0.209	0.04	1.27E-08
rs11717223	Intron	3	186725404	A	G	0.20	0.98	0.210	0.04	1.27E-08
rs4686842	Intron	3	186761377	G	A	0.27	0.94	-0.201	0.04	1.30E-08
rs3868902	Intron	3	186715887	G	T	0.30	0.96	-0.197	0.03	1.31E-08
rs113622634	Intron	3	186718076	G	A	0.21	1.00	0.209	0.04	1.40E-08
rs3887352	Intron	3	186720916	G	T	0.21	1.00	0.209	0.04	1.50E-08
rs16861432	Intron	3	186719041	A	G	0.21	1.00	0.208	0.04	1.52E-08
rs3887353	Intron	3	186721192	C	T	0.21	0.99	0.208	0.04	1.53E-08
rs11711797	Intron	3	186718722	T	C	0.21	1.00	0.208	0.04	1.54E-08
rs6799007	Intron	3	186719705	C	T	0.21	1.00	0.208	0.04	1.59E-08
rs6444188	Intron	3	186677936	G	T	0.38	0.98	0.183	0.03	1.71E-08
rs16861424	Intron	3	186717963	A	G	0.21	0.99	0.207	0.04	1.91E-08
rs11711828	Intron	3	186718798	T	C	0.21	0.99	0.207	0.04	1.91E-08
rs16861497	Intron	3	186731327	G	T	0.43	0.97	0.176	0.03	2.13E-08
rs12632862	Intron	3	186669214	A	G	0.45	0.99	0.174	0.03	2.31E-08

rs4686811	Intergenic variant	3	186617522	G	C	0.42	0.93	0.179	0.03	2.77E-08
rs2889790	Intron	3	186717475	T	G	0.21	0.99	0.207	0.04	2.84E-08
rs112277894	Intron	3	186730302	A	T	0.21	0.98	0.209	0.04	3.19E-08
rs6788832	Non coding transcript exon variant	3	186724219	A	G	0.33	0.99	-0.180	0.03	3.42E-08
rs13322676	Intron	3	186718745	A	C	0.26	0.99	-0.198	0.04	4.02E-08
rs13058859	Intron	3	186676537	C	T	0.43	0.98	0.173	0.03	4.08E-08
rs6780488	Intron	3	186763942	G	A	0.21	0.93	-0.214	0.04	4.41E-08
